# Performance of a Three-Dimensional Electrochemical Reactor (3DER) on Bisphenol A Degradation

**DOI:** 10.3389/fchem.2022.960003

**Published:** 2022-07-15

**Authors:** Xu Ren, Kai Song, Qiaoyun Zhang, Linghan Xu, Zhuyi Yu, Peixin Tang, Zhicheng Pan

**Affiliations:** ^1^ Sichuan Provincial Engineering Research Center of City Solid Waste Energy and Building Materials Conversion and Utilization Technology, Chengdu University, Chengdu, China; ^2^ Postdoctoral Research Station of Haitian Water Group CO, Ltd, AVIC International Exchange Center, Chengdu, China; ^3^ Postdoctoral Research Station in Environmental Science and Engineering, Sichuan University, Chengdu, China; ^4^ Faculty of Geosciences and Environmental Engineering, Southwest Jiaotong University, Chengdu, China

**Keywords:** three-dimensional electrochemical reactor (3DER), advanced oxidation, bisphenol A (BPA), removal, pathway

## Abstract

This study constructed a three-dimensional electrochemical reactor (3DER) using meshed stainless steel sheets and titanic magnetite particles (TMP) to investigate bisphenol A (BPA) degradation through the synergistic action of electrical current and TMP. We examined some TMP characteristics, such as particle size, specific surface areas, X-ray diffraction, surface imaging, elemental constituents, and electrical resistivity. It was found that TMP was a micron-level material with excellent electrical conductivity, and it could be regarded as a magnetite-based material comprising Fe(II) and Fe(III). The single-factor experiment determined the optimal conditions for BPA removal in 3DER, specifically by introducing 200 ml of BPA-simulated wastewater (10 mg L^−1^) into 3DER. At the initial pH of 9.00, current and electrodes gap of 300 mA and 15 mm, respectively, and adding 1 ml of 0.5 M potassium peroxymonosulfate and 1 g TMP, > 98% of BPA was removed after 55 min of electrochemical reaction. In addition, liquid chromatography–mass spectrometry identified the intermediates formed during the BPA treatment, showing two possible pathways for BPA degradation. The final degradation intermediates were chain organics with simple molecular structures. This research provided an understanding of the potential application of 3DER for BPA removal in water.

## 1 Introduction

Bisphenol A (BPA) is a typical emerging contaminant found in surface, ground, and waste waters (even in tap water), causing endocrine disorders (Huang and Huang, 2009; Xu et al., 2019a). However, BPA is still one of the most widely used industrial compounds globally, as the global demand for BPA-based products could grow at a 4.7% annual rate through 2030 (Kakavandi et al., 2022). Although BPA is currently detected in low environmental concentrations, it poses a severe health threat to humans and animals due to its high toxicity and extreme stability in water (Ji et al., 2018; Jing et al., 2021). Therefore, it is critical to effectively control environmental BPA levels to ensure human health and ecological safety.

Advanced oxidation technologies (AOTs) involving reactive oxygen species (ROS), such as hydroxyl radical (HO•), sulfate radical (SO_4_^•−^), chlorine radicals (e.g., Cl), and hypochlorite (•ClO^−^), have been considered an effective technology for treating emerging contaminants in water (Xu et al., 2019b; Zhi et al., 2020; Wang et al., 2021). Therein, AOTs of organics based on SO4^−^ oxidation have received massive attention in scientific research and industrial applications. On the one hand, persulfates (PS), including peroxydisulfate (PDS) and peroxymonosulfate (PMS), are a common source of SO_4_^•−^, O_2_, O_2_; they are relatively stable oxidants, easily transportable, and storable (Chen and Huang, 2015; Kiejza et al., 2021). Also, it is non-selective for oxidizing organics in a wide pH range (2–8) and has a similar redox potential (E^o^ = 2.5–3.1 V vs. NHE) with HO (E^o^ = 2.74 V vs. NHE) (Ledjeri et al., 2016; Peng et al., 2021). SO_4_^−^ could be produced *via* heat activation, UV light activation, metal activation, electrolytic activation, and a combination of these methods (Sharma et al., 2015; Ren et al., 2021; Wei et al., 2021).

Electrochemical advanced oxidation technologies (EAOTs) have been regarded as promising AOT with several advantages, such as environmental friendliness, high contaminants removal efficiencies, and easy operation (Chen et al., 2017; Ren et al., 2020; Lu, 2021). Nevertheless, conventional EAOTs [e.g., two-dimensional electrochemical reactors (2DERs)] still have some drawbacks, such as relatively low current efficiency and small treatment capacity, limiting their applications (Nidheesh et al., 2020; Quang et al., 2022).

Three-dimensional electrochemical reactors (3DERs) have become a hot topic in recent decades (Li et al., 2019a; Ni et al., 2020; Ma et al., 2021). A 3DER was established based on 2DER by filling conductive particles between the parallel (or 2D) electrodes (Cho et al., 2020; Ma et al., 2021). The electrical conductive particles are also called particle electrodes (PEs), third electrodes, or bed electrodes (Zhang et al., 2013). In the electric field, PEs are polarized to form several microelectrodes with different charged ends (Zhang et al., 2013; Shi et al., 2020; Zhou et al., 2021), that is, anodic and cathodic surfaces. This process illustrates that electrochemical reactions occur on the electrodes surfaces and PE ends. Compared with 2DER, 3DER can increase the electrode reaction area and mass transfer efficiency, thereby improving pollutant removal efficiency (Zhang et al., 2013).

Therefore, if PS is used as the electrolyte and PEs are the metallic materials with catalytic and conductive abilities, the 3DER could be regarded as an advanced oxidation system coordinated with metal-PS (a heterogeneous catalytic system) electro-PS system (Li et al., 2019b). Therefore, in this study, the 3DER was constructed by 2D electrodes using meshed stainless steel sheets and titanic magnetite particles (TMP), which are iron-based materials with catalytic performance and excellent electrical conductivity according to the previous study. Moreover, PMS was used as the oxidant and electrolyte.

We investigated the performance of 3DER degradation of BPA under various conditions (including oxidant concentration, current, gap of 2D electrodes, and some inorganic ions) in four control experiments to determine the optimal condition. In addition, the possible BPA degradation pathway in 3DER was analyzed by monitoring the intermediate products.

## 2 Methods and Materials

### 2.1 Chemicals and Setup

Bisphenol A (BPA, C_15_H_16_O_2_, CAS 80-05-7), with a molecular weight of 228 and >99.0% purity, was purchased from Aladdin and used as received. Other chemicals, including potassium peroxymonosulfate (PMS, 2(KHSO_5_) KHSO_4_ K_2_SO_4_; H_3_K_5_O_18_S_4_), sodium thiosulfate (Na_2_O_3_S_2_), sodium hydroxide (NaOH), and sulfate acid (H_2_SO_4_) were procured from Chron Chemicals company (Sichuan Province, China). All the chemical reagents employed in this study were analytical grade or guaranteed.

The TMP samples were collected from a vanadium–titanium–magnetite concentrating plant in China’s Pan Xi region of Sichuan Province. The samples were rinsed thrice with ultrapure water (resistance of 18.25 MΩ cm^−1^) and dried at 35°C before use.

### 2.2 Experiment Procedure

**FIGURE 1:**
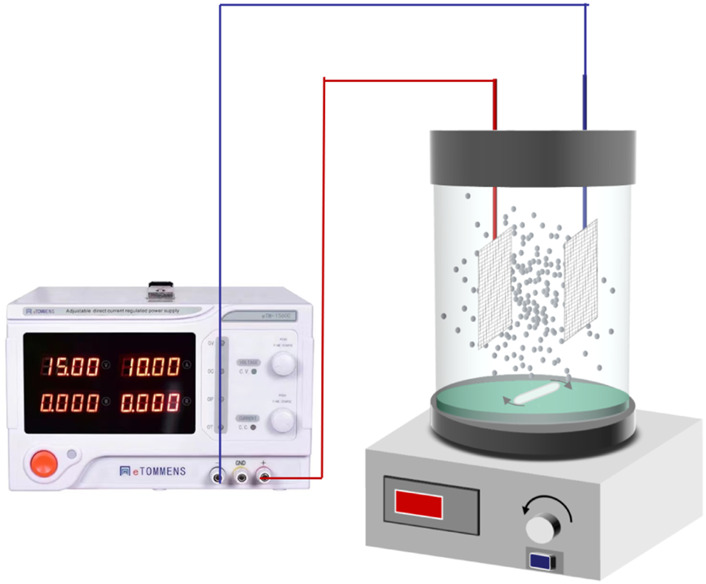
Schematic of the experimental setup.

The main units of the experimental setup (Figure 1) were a constant current power supply (TPR-1520D, Longwei Instrumentation Co. LTD., Hong Kong, China), magnetic stirrers (MS-D6, Hu Xi Co. LTD., Shanghai, China), a 250 ml electrolysis cell made of organic glass, TMP (as PEs), and two mesh stainless steel sheets (35 mm × 25 mm × 1 mm) as 2D electrodes.

Certain PE amounts were added into the electrolysis cell bearing 200 ml of 10 mg L^−1^ BPA. The mixture was stirred for 15 min at 1,000 rpm to ensure homogenization. Then the predetermined volume of PMS solution (0.5 mol L^−1^) was injected into the electrolysis cell, and the power was turned on to start the reaction. The effect of current, PMS concentration, PEs dosage, 2D electrodes gap, and initial pH was investigated successively *via* a single-factor experiment. The initial pH of the BPA simulated wastewater was adjusted with 1 M H_2_SO_4_ and 5 M NaOH before the electrochemical reaction. The samples were collected at 0, 5, 10, 15, 20, 30, 40, and 55 min and filtered through 0.22-μm filter films to analyze the BPA concentration. All tests were conducted in duplicates, and the standard deviations are reported to minimize experimental errors.

### 2.3 Analytical Methods

The BPA concentration was determined using ultra-performance liquid chromatography (UPLC, 1,290 Infinity, Agilent, United States) equipped with a Spheri-5 C18 column (150 mg/L × 4.6 mg/L, 5 μm) and a UV detector (276 nm for BPA). Acetonitrile and ultrapure water (50:50) were applied as the mobile phase, and the analytical scan range was 90–400 nm at a 2 mm interval. The intermediate products of BPA degradation were determined by liquid chromatography–mass spectrometry (LC-MS) using the electrospray ionization positive ion mode as the ionization source. The mobile phase was the same as UPLC, while the flow rate and m/z scanning range were 0.2 ml/min and 100–600, respectively. Free radicals generated in the 3DER were detected by electron paramagnetic resonance (EPR, Bruker EMXPlus-10/12, Germany). Here, DMPO (5, 5-dimethyl-1-pyrrolidine N-oxide) as a spin-trapping agent was used in the EPR measurement. The mixture of standard EPR spin trapping experiment contains 100 mm DMPO, 1.25 mm PMS, and 5.0 g/L TMP in ultrapure water with electrical current of 300 mA and 2D electrodes gap of 15 mm.

The TMP particle size distribution was detected by a laser particle size meter (Mastersizer 3,000, Malvern, United Kingdom). In addition, Micromeritics ASAP 2460 (Micromeritics, United States) analyzed the nitrogen gas uptake isotherms to calculate the specific surface area based on the Brunauer–Emmett–Teller (BET) model. Furthermore, X-ray diffraction (XRD, XD-2, Pu Xi, China) assessed the chemical constituents of the PEs. The two theta (2θ) diffraction within 10°–70° and at 0.02° interval was examined at a 4°min^−1^ scanning speed. The microstructure and elemental content of TMP were investigated by scanning electron microscopy and energy dispersive spectroscopy (SEM-EDS, JSM-5900LV, JEOL, Japan), working at 25 kV and 30 nm resolution. In addition, the electrical resistivity of the TMP sample was detected *via* a semiconductor powder resistivity tester (ST-2722, Suzhou Jing Ge, China) using a four-probe method. Before the test, TMP was made into 10 mm × 10 mm × 0.11 mm sheets.

For data processing, the C/C_0_ values represent the ratio of BPA concentration after a certain reaction time to the initial BPA concentration (10 mg L^−1^). Therefore, the removal rate of BPA could be represented as Eq. 1. Plotting was done using Origin 2022.

$$\mathrm{BPA}\;removal\;rate=\left(1-C/C_{0}\right)\times 100\%.$$ (1)

## 3 Results and Discussion

### 3.1 Characteristics of TMP

**FIGURE 2:**
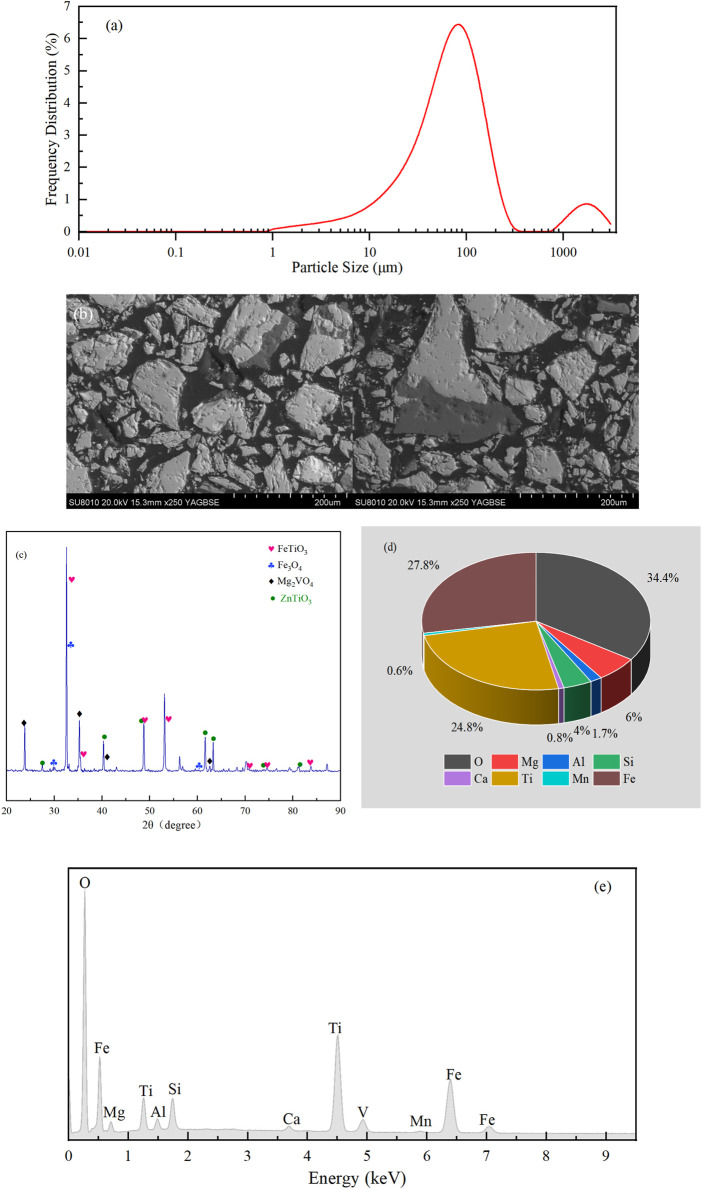
Characteristics of PEs: **(A)** particle size distribution; **(B)** SEM images; **(C)** XRD results; **(D)** EDS results; **(E)** EDS element spectrum.

The TMP is a micron-grade material whose particle sizes are predominantly between 10 and 300 µm (Figure 2A). The SEM images (Figure 2B) showed that the particles had irregular shapes with relatively rough surfaces. Furthermore, the BET and Langmuir specific surface areas were −0.259 and 0.053 m^2^/g, respectively. Such relatively small specific surface areas suggested that BPA adsorption could be ignored during the reaction. The XRD analysis (Figure 2C) showed that ilmenite (FeTiO_3_, 2θ = 35.26^o^, 48.74^o^, and 53.10^o^) and magnetite (Fe_3_O_4_, 2θ = 30.01^o^, 35.50^o^, and 60.32^o^) were the main constituents of Pes. The materials also contained Mg_2_VO_4_ and ZnTiO_3_. Moreover, the EDS results revealed 34.4% of O, 27.8% of Fe and 24.8% of Ti in PEs (Figures 2D,E). The average electrical resistivity of PEs was 6.29 *Ω* cm × 10^3^ *Ω* cm, showing that TMP has excellent conductivity, which is consistent with the study of Zhong et al. (2012). Generally, TMP is a micron-level material with excellent conductivity and catalytic ability. Hence, it can be regarded as a magnetite-based material bearing Fe(II) and Fe(III) (Liang et al., 2010; Fang et al., 2017; Lai et al., 2020). Consequently, TMP can be used as PEs to construct 3DER along with 2D electrodes.

### 3.2 Factors Affecting BPA Removal

#### 3.2.1 Electrical Current

**FIGURE 3:**
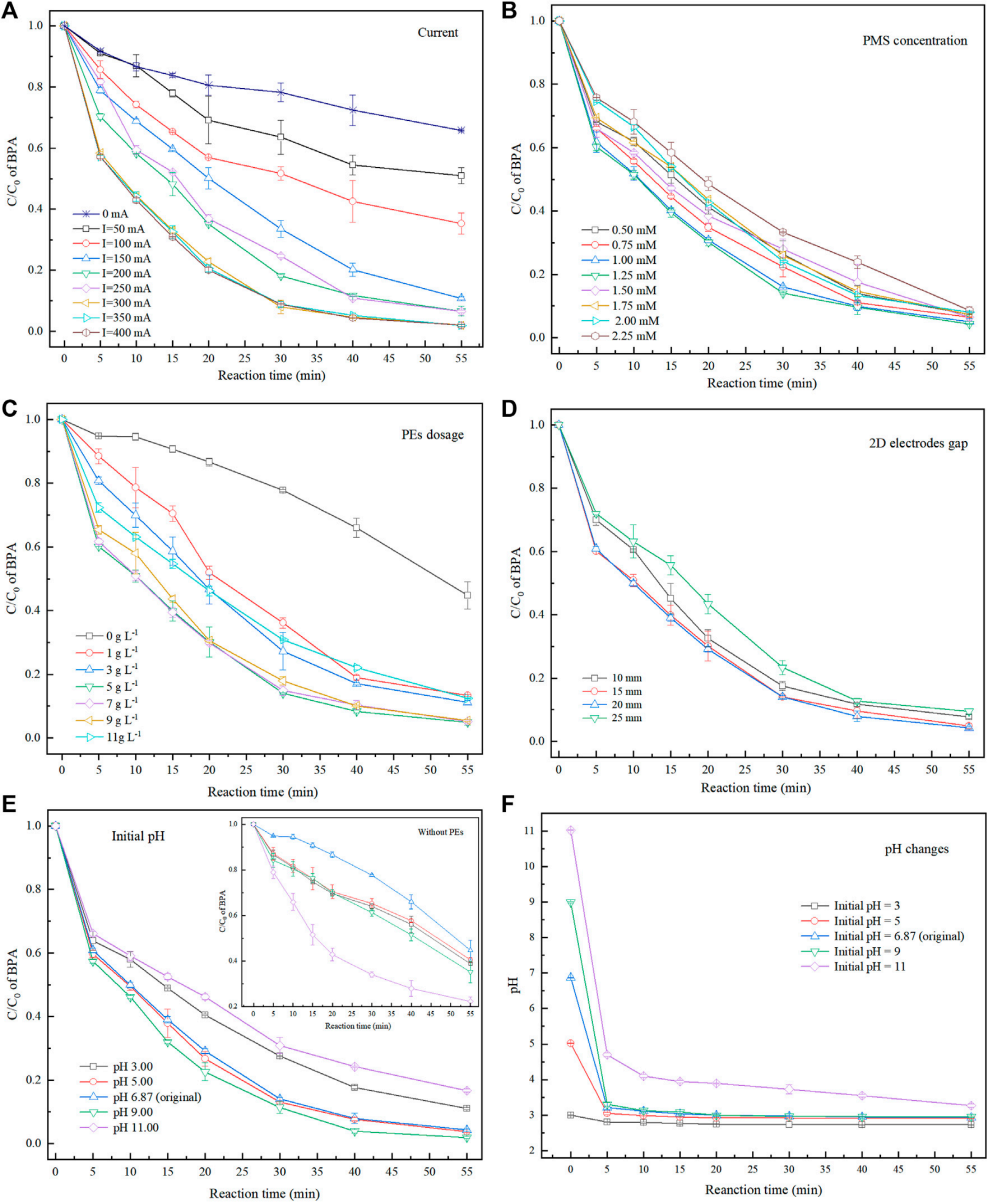
Degradation of BPA under various conditions: **(A)** current; **(B)** PMS concentrations; **(C)** PEs dosage; **(D)** 2D electrodes gap; **(F)** initial pH; **(G)** system pH changes during electrochemical reaction.

Electrical current, an essential factor affecting the electrochemical treatment efficiency, determines the amount of electron transfer in the reactor (Guo et al., 2022). Therefore, the effect of current on BPA removals was researched when the PMS concentration, PEs dosage, and 2D electrodes gap were 1 mm, 5 g L^−1^, and 15 mm, respectively, the initial pH was unadjusted and the results were shown in Figure 3A. After 55 min of electrochemical reaction under 50, 100, 150, 200, 250, 300, 350, and 400 mA currents, the removal rates of BPA were 60.75, 80.21, 89.21, 93.38, 95.49, 98.00, 98.07, and 98.03%, respectively. BPA removal increased notably with the applied current from 50 to 300 mA, while further increase showed less impact. Moreover, when the current was 0 mA, the BPA removal reached 37.72% after 55 min of reaction. Previous studies showed that magnetite or magnetite-based materials are ideal catalysts, capable of activating some oxidants (e.g., H_2_O_2_ and PS) into ROS [including HO•, SO_4_^•−^, O_2_^•−^, and Fe(IV)], enabling BPA degradation *via* their oxidation (Eq. 2).

Promoting BPA removal by increasing the electrical current could be ascribed to the following reasons: PMS was activated under the synergistic action of current and PEs, and the system produces more ROS (Eqs. 3–5), which can decompose the BPA *via* a process called indirect oxidation (Eq. 2); For another, BPA can be directly mineralized (oxidized) on the electrodes (Eq. 6) (Cho et al., 2020; Tang et al., 2021). Overall, as the current increased, the redox reaction at 2D electrode surface was promoted at the anodic and cathodic PE surfaces. However, excessive current may lead to electrode passivation (Wu et al., 2019; Ren et al., 2020), causing the BPA removal to retard. Therefore, the optimal current for the 3DER should be set to 300 mA.

$$\mathrm{BPA}+ROS\to \mathrm{intermediates}\to CO_{2}\left(g\right)+H_{2}O\left(aq\right),$$ (2)

$$HSO_{5}^{-}+e^{-}\to SO_{4}^{•-}{+}^{•}OH,$$ (3)

$$HSO_{5}^{-}+M^{n+}\to SO_{4}^{•}+M^{\left(n+1\right)+}+OH^{-},$$ (4)

$$M^{\left(n+1\right)+}+e^{-}\to M^{n+},$$ (5)

$$BPA+e^{-}\to CO_{2}+H_{2}O.$$ (6)

In Eqs 4, 5, M represents the transition metal irons in PEs, such as Fe(II) and Mn(II).

#### 3.2.2 PMS Concentration

The role of PMS is crucial to the electrochemical reaction because it serves as an oxidant and electrolyte. The effect of initial PMS concentrations (0.5–2.25 mm) on BPA removal was studied, when the current was 300 mA, PEs dosage was 5 g L^−1^, the gap of 2D electrodes was 15 mm and initial pH was unadjusted. It can be seen in Figure 3B, the BPA removal improved with PMS concentration (0.5–1.25 mm), reaching 95.64% after 55 min of reaction with 1.25 mm PMS concentration. More PMS means more sources of ROS, resulting in stronger indirect oxidation of the system. Meanwhile, increasing the PMS concentration could improve the electron transfer efficiency of the system. However, with a further increase in PMS concentration, BPA removal decreased. These phenomena could be attributed to the following reasons: first, a given amount of PEs could only provide certain number of active reaction sites to activate PMS. Similarly, only a certain amount of PMS can be activated under a given electric current. Second, excess PMS could produce some side reactions (Eqs. 7,8) (Tang et al., 2021), decreasing the SO_4_^•−^ (with higher redox potential) than SO_5_^•−^ (E^0^ = 0.81 V vs. NHE) and S_2_O_8_^2−^ (E^0^ = 2.01 V vs. NHE) (Xia et al., 2020) in the system. Furthermore, the degradation effect *via* direct oxidation of BPA is limited by electrical current. Accordingly, the optimal PMS concentration for BPA degradation was 1.25 mm.

$$SO_{4}^{•-}+HSO_{5}^{-}\to +SO_{5}^{•-}+HSO_{4}^{-},$$ (7)

$$SO_{4}^{•-}+SO_{4}^{•-}\to S_{2}O_{8}^{2-}.$$ (8)

#### 3.2.3 PE Dosage

The effect of PEs dosage (from 0 g L^−1^–11 g L^−1^) in the 3DER on BPA degradation is shown in Figure 3C. Other experiment conditions, such as electrical current, PMS dosage, and the 2D electrodes gap was 300 mA, 1.25, and 15 mm, respectively, and the initial pH was unadjusted. The BPA removal rate was only 55.26% after 55 min contact without PEs addition. However, it could reach 86.64% with only 0.2 g PE (1 g L^−1^) in the system. Furthermore, the BPA removal peaked at 95.06% with a 5 g L^−1^ PEs dosage after 55 min of treatment. Increased dosage to 7 g L^−1^ was insignificant, while BPA removal decreased when PEs dosage was increased to 11 g L^−1^.

The PEs were polarized, and several microelectrodes were formed in the electric field. Nevertheless, too many particles could cause an easy collision with each other, resulting in a short circuit and agglomeration due to electrical adsorption, which may lead to decreased electrochemical reaction area. Consequently, keeping the PEs dosage of 5 g L^−1^ was the optimum preferred.

#### 3.2.4 The 2D Electrodes Gap

The 2D electrodes gap is another essential factor affecting the performance of an electrochemical reactor. Here, we investigated the effect of different 2D electrodes gaps (10, 15, 20, and 25 mm) on the BPA removal rates (Figure 3D). Other experiment conditions, such as electrical current, PMS dosage and PEs dosage was 300 mA, 1.25 mm and 5 g L^−1^, respectively, and the initial pH was unadjusted. The 3DER performance was relatively poor under 10 and 25 mm 2D electrode gaps. The too-short distances accelerate electrode passivation and shorten the diffusion distance of ROS produced by the electrodes (Guo et al., 2021). In addition, fewer PEs between the electrodes with a shorter 2D electrodes gap ensued, hence the less synergistic action of current and PEs. Although more particles existed between the 2D electrodes at longer distances, the current density between the electrodes decreased significantly. Moreover, too large 2D electrode gap would increase the IR-drop, increasing the average voltage ( $\bar{U}$ refers to the average of the initial and final voltages) and energy consumption (*E*) of the system (Eq. 9) (Ren et al., 2021) when the current (*I*) and reaction time (*T*) are constant. In this case, $\bar{U}$ was 44.81 V when the 2D electrode gap was 25 mm, while this value for 15 and 20 mm was 25.95 and 37.41 V, respectively. Therefore, considering the treatment effect and energy consumption comprehensively, it was determined that 15 mm was the optimal 2D electrodes gap; here, the BPA removal rate reached 95.06% at 55 min.

$$E=\bar{U}\times I\times T.$$ (9)

#### 3.2.5 Initial pH

The initial solution pH affects the electrochemical and heterogeneous catalytic processes, including types of ROS, catalyst (i.e., PEs) surface properties, and the degree of organic hydrolysis. The experiments were conducted when electrical current, PMS dosage and PEs dosage was 300 mA, 1.25 mm and 5 g L^−1^, respectively. Figure 3E presents the effect of various initial pH on BPA removal. Of note, there was no buffer introduced in the system, as their components, e.g., phosphates and carbonates might affect the BPA removal. The results indicated that the relatively outstanding BPA removal performance (the removal rate was 98.1% at 55 min) was reached at pH 9.00. Due to PMS hydrolysis (Eqs. 10,11), the initial pH values (3.00, 5.00, 6.87, 9.00, and 11.00) of electrolyte rapidly decreased to 2.81, 3.01, 3.21, 3.31, and 4.70, respectively in the initial 5 min. The values finally changed to a similar pH (2.70–3.30) after 55 min reaction (Figure 3F).

Many studies have reported that SO_4_^•−^ is the dominant ROS at pH < 7, while the neutral-to-alkaline conditions could promote the SO_4_^•−^ transformation to ROS with relatively high redox potential, such as HO•, O_2_^•−^, and 1O_2_ (Eqs. 12–16) (Li et al., 2019b). The improvement effect on the treating emerging contaminants could be observed at pH 6—11. These trends could also be confirmed in controlled experiments without PEs (Figure 3E). The treatment effect at pH 11 is better than in other pH conditions. However, BPA removal decreased sharply as the initial pH rose from 9 to 11 in 3DER. Many flocs were observed in the 3DER during the reaction. These phenomena could be ascribed to the formation of Fe(OH)_n_ (*n* = 2, 3) colloids by Fe(II) and Fe(III) in the PE reactions with OH^−^ (under the action of current), thereby decreasing the ROS concentrations in the system. Therefore, for 3DER, an initial pH of 9.00 was the optimal condition for BPA removal.

$$HSO_{5}^{-}\to H^{+}+SO_{5}^{2-},$$ (10)

$$HSO_{5}^{-}+H_{2}O\to H_{2}O_{2}+HSO_{4}^{-},$$ (11)

$$SO_{4}^{•-}+H_{2}O\to SO_{4}^{2-}+H^{+},$$ (12)

$$SO_{4}^{•-}+OH^{-}\to SO_{4}^{2-}+HO^{•},$$ (13)

$$HO^{•}+H_{2}O_{2}\to HO_{2}^{•},$$ (14)

$$HO_{2}^{•}\to H^{+}+O_{2}^{•-},$$ (15)

$$O_{2}^{•-}+H_{2}O_{2}\to O_{2}+HO^{•}+OH^{-}.$$ (16)

Conclusively, the optimal conditions included introducing 200 ml of 10 mg L^−1^ BPA-simulated wastewater with 1 g TMP into the 3DER, stirring for 30 min at 1,000 rpm, and adjusting the initial pH to 9.00. In addition, the current and electrode gap were set at 300 mA and 15 mm, respectively, thereafter 1 ml of 0.5 M PMS was injected into the reactor, allowing the process to lapse 55 min.

The comparison of the optimal conditions for the typical refractory organics removal by 3DER in this study with those reported in other literatures recently is listed in Table 1. In general, TMP is a natural material that is more economical than many synthetic materials used as PEs in 3DER.

**TABLE 1:** Optimal conditions for the typical refractory organics removal by 3DER.

Contaminate	Materials of 2D electrodes materials	PEs materials	Optimal conditions	Reaction time (min)	Contaminate removal rate	References
Atrazine (10 mg L^−1^, 400 ml)	Anode: Ti/RuO_2_-IrO_2_; Cathode: stainless steel	CuFe_2_O_4_	CD = 4 mA/cm^2^; PDS dosage = 4.0 mM; PEs dosage = 3.0 g L^−1^; initial pH = 6.30	60 min	>99.00%	Li et al. (2019b)
Amoxicillin (200 mg L^−1^, 500 ml)	Anode: Ti/RuO_2_; Cathode: Ti/RuO_2_	Granular active carbon (GAC) and quartz sand (9:1)	CD = 5 mA/cm^2^; PEs volume = 50 cm^3^; initial pH = 5.56	120 min	98.98%	Shi et al. (2020)
Rhodamine B (1,000 mg L^−1^, 1,500 ml)	Anode: Pb/PbO2,; Cathode: stainless steel	GAC	CD = 23 mA/cm^2^; 2D electrodes gap = 30 mm; pH = 7.6	60 min	97.4%	Shokoohi et al. (2020)
BPA (20 mg L^−1^, 100 ml)	Anodes: Ti/RuO_2_-IrO_2_; Cathode: GDE	Fe_3_O_4_/N-rGO	CD = 6 mA/cm^2^; 2D electrodes gap = 25 mm; PEs dosage = 0.1 g L^−1^; initial pH = 3.0	90 min	90.00%	Zhang et al. (2020)
Tetracycline (25 mg L^−1^, 100 ml)	Anode: Pt; Cathode: graphite	MnFe_2_O_4_	CD = 30 mA/cm^2^; PDS dosage = 3.0 mM; PEs dosage = 0.3 g L^−1^; 2D electrodes gap = 20 mm; initial pH = 3.00	60 min	86.23%	Tang et al. (2021)
4-Chlorophenol (500 mg L^−1^, 300 ml)	Anodes: Ti/RuO_2_-IrO_2_; Cathode: Ti	Biochar-loaded material	Current = 1 A; 2D electrodes gap = 30 mm; PEs dosage = 16.67 g L^−1^; Na_2_SO_4_ = 2 g/L	150 min	99.93%	Xie et al. (2021)
Berberine (14 mg L^−1^, 100 ml)	Anode: Ti/RuO_2_-IrO_2_; Cathode: gas diffusion electrode (GDE)	Fe_3_O_4_/SnO_2_/GO	Current density (CD) = 15 mA/cm^2^; PEs dosage = 0.2 g L^−1^; 2D electrodes gap = 30 mm; initial pH = 3.00	120 min	71.70%	Zhou et al. (2021)

### 3.3 Identification of Main Free Radicals

**FIGURE 4:**
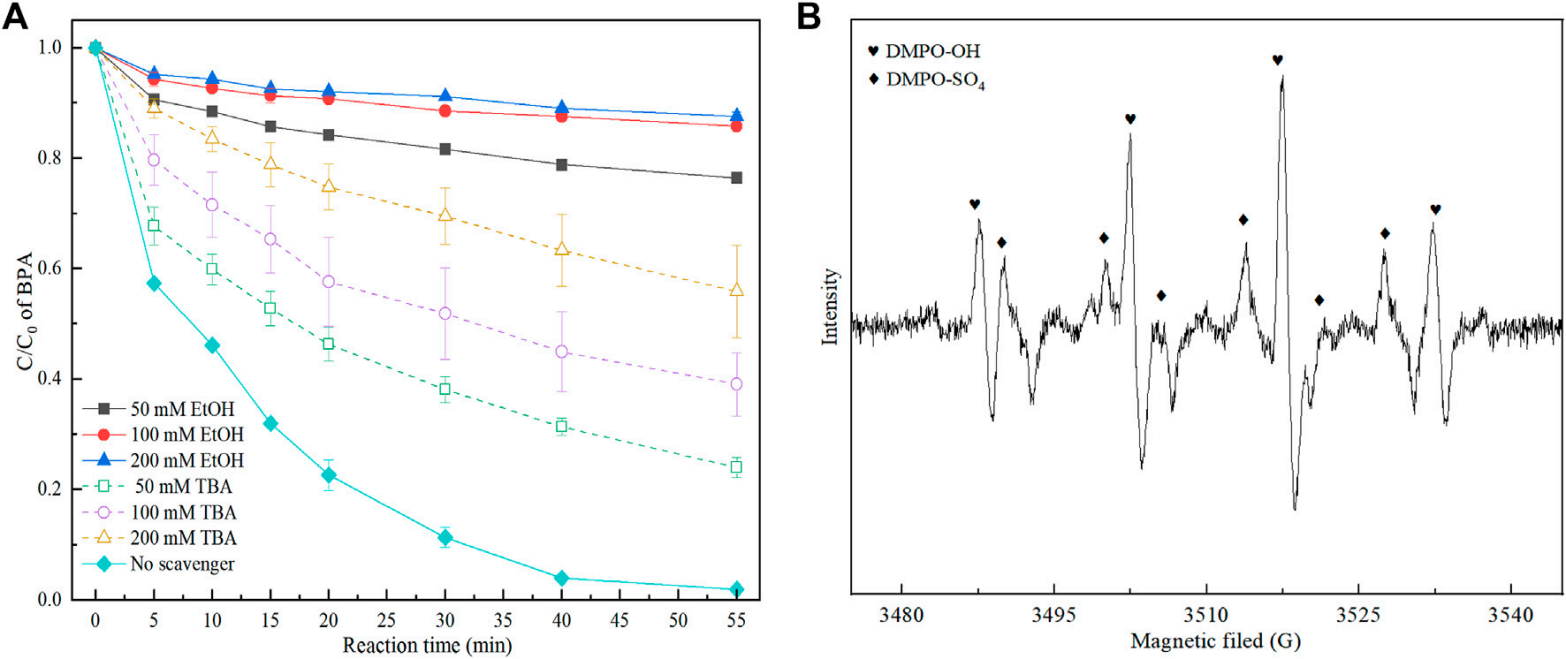
Effect of EtOH and TBA on the BPA removals **(A)** and the EPR spectra in 3DER **(B)**.

It has been well documented that when PMS was activate by electrical current or/and transition metal-based materials in the aqueous solution, it always formed free radicals including SO_4_^•−^ and HO•. In this study, tert-butyl alcohol (TBA) was used as the HO• scavenger, while ethyl alcohol (EtOH) was used as a quenching agent for both SO_4_^•−^ and HO•. It can be seen from Figure 4A, the BPA removal rates were decreased with the increase of the addition of scavengers, suggesting that both SO_4_^•−^ and HO• formed in 3DER. And extremely low BPA degradation efficiencies (about 23.6,14.2 and 12.4%, respectively) were obtained with addition of 50, 100 and 200 mm EtOH. However, the BPA removal rates (76.0, 60.9, and 44.1%) were higher when the same amount of TBA was added in 3DER. Obviously, the inhibition effect of EtOH was much higher than TBA, illustrating the SO_4_^•−^ should account for a larger proportion in the 3DER. Meanwhile, the EPR experiment in 3DER was conducted with DMPO, which was used as a trapping agent to analyze the dominant ROS. Figure 4B shown the characteristic peaks were the signal of DMPO-OH and DMPO-SO_4._ These phenomenon indicated original SO_4_^•−^ could convert into HO•, and work together to degrade BPA.

### 3.4 Stability of 3DER

**FIGURE 5:**
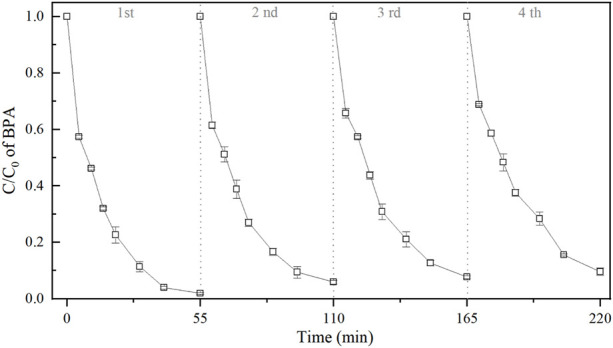
Results of cyclic experiments.

To examine the stability of the 3DER cyclic experiments were performed for four cycles. The PEs were separated from reaction system after each run by an applied magnetic force, and ultrasonic cleaned for 20 min to remove adsorbents on their surface, such as sulfate deposits. Following dried recovered PEs to constant weight at 35°C, and the agglomerates were ground into fine particles. Figure 5 shows that the BPA removals decreased with the increase of use time. Compared with the BPA removal efficiency at 55 min of reaction time (the first cyclic experiment), it decreased by 7.7% when the reaction time was 220 min (the fourth cyclic experiment). More than 90% of BPA were removed after each run, indicating an excellent recyclability of PEs.

In addition, in order to realize practical application of the 3DER, the processing costs of 3DER are an issue to be considered. Therefore, we chose the stainless steel electrode with low cost. Although the anode dissolved continuously, it dissolved extremely slowly at low current conditions. We ran about 100 experiments with the same pair of 2D electrodes and found that the electrodes were still functional, but needed to be cleaned regularly of the oxides on their surface. In general, the stability of 3EDR is somewhat excellent.

### 3.5 Pathway of BPA Degradation in 3DER

**FIGURE 6:**
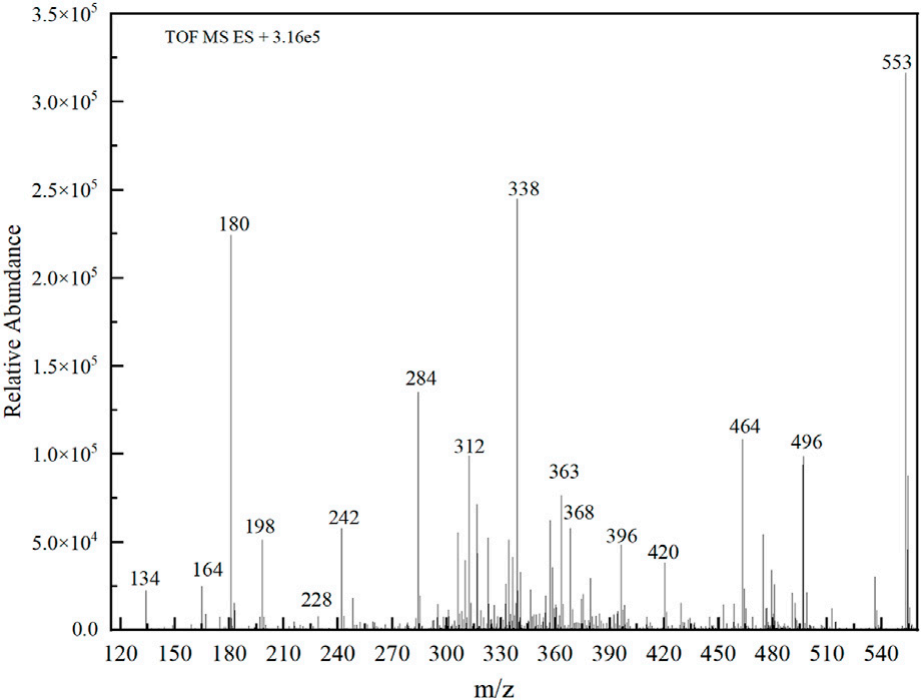
MS spectra of the intermediates.

The PMS was activated by the synergy of current and PEs. The ROS reacted with aromatic ring and C=C bonds of BPA, thereby forming intermediates, such as radical sulfate adducts and hydroxylation products. To investigate the pathway of BPA degradation in 3DER, LC-MS analyzed the degradation products. The results (Figure 6) identified several product peaks with lower molecular weight than BPA, including the maximum mass to charge ratio (m/z) of 134, 164, 180, and 198. Meanwhile, the products with relatively high molecular weight were detected at m/z of 242, 284, 338, and 553. Nonetheless, some intermediates were hardly detected for their concise life span. For instance, the short life of sulfate radical adducts was usually <200 ns. Compared with previous studies, likely BPA degradation intermediates are listed in Table 2.

**TABLE 2:** Possible products of BPA degradation.

Item	Maximum m/z	Molecular formula	Possible Structure	Detected or reported Previous
1	228	C_15_H_16_O_2_	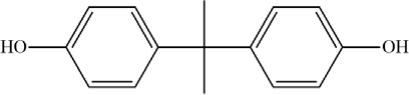	Detected
2	553	C_37_H_44_O_4_	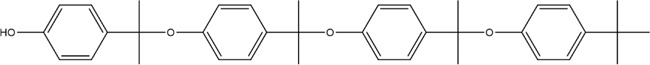	Detected
3	496	C_33_H_36_O_4_	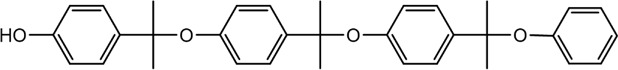	Detected
4	463	C_30_H_38_O_4_	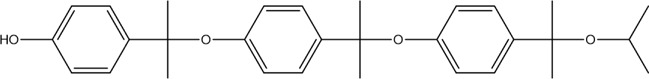	Detected
5	420	C_27_H_32_O_4_	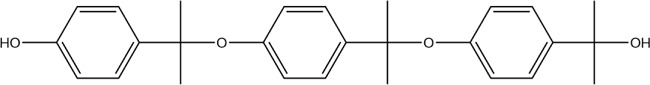	Detected
6	312	C_15_H_20_O_7_	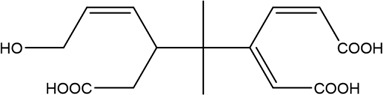	Detected
7	284	C_14_H_20_O_6_	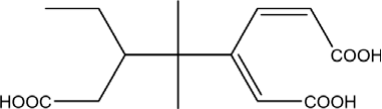	Detected
8	260	C_15_H_16_O_4_	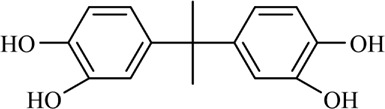	Wen et al., 2015
				Lin et al. (2013)
9	256	C_15_H_12_O_4_	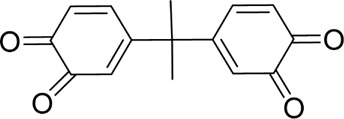	Du et al., 2016
				Lin et al. (2020)
10	248	C_14_H_16_O_4_	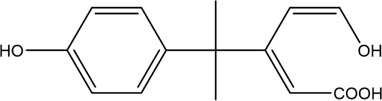	Detected
11	244	C_15_H_16_O_3_	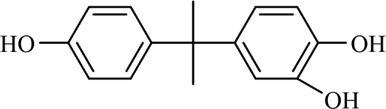	Samarghandi et al. (2021)
12	242	C_15_H_14_O_3_	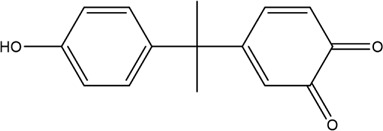	Detected
13	198	C_10_H_14_O_4_	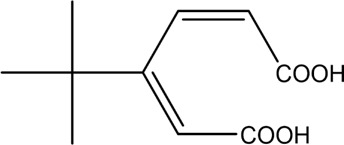	Detected
14	180	C_10_H_12_O_3_	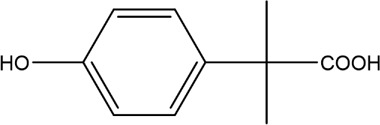	Detected
15	164	C_10_H_12_O_2_	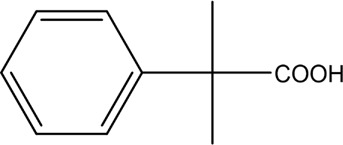	Detected
16	152	C_9_H_12_O_2_	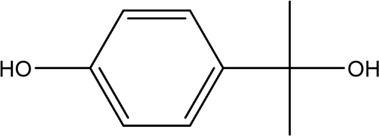	Ji et al. (2018)
17	136	C_9_H_12_O	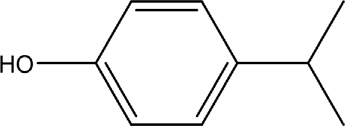	Lin et al. (2020)
18	136	C_9_H_12_O	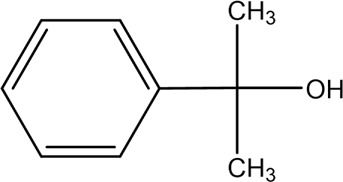	Lin and Zhang., 2017
19	130	C_6_H_10_O	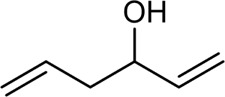	Lin et al. (2020)
20	134	C_9_H_10_O	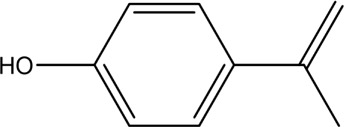	Detected
21	118	C_4_H_6_O_4_	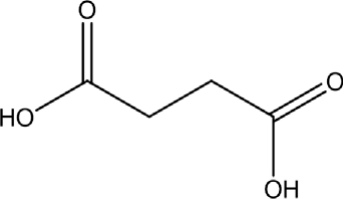	Olmez-Hanci et al. (2013)
22	108	C_6_H_4_O_2_	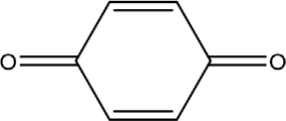	Ji et al. (2018)
23	94	C_6_H_6_O	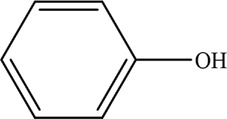	Lin et al. (2020)

**FIGURE 7:**
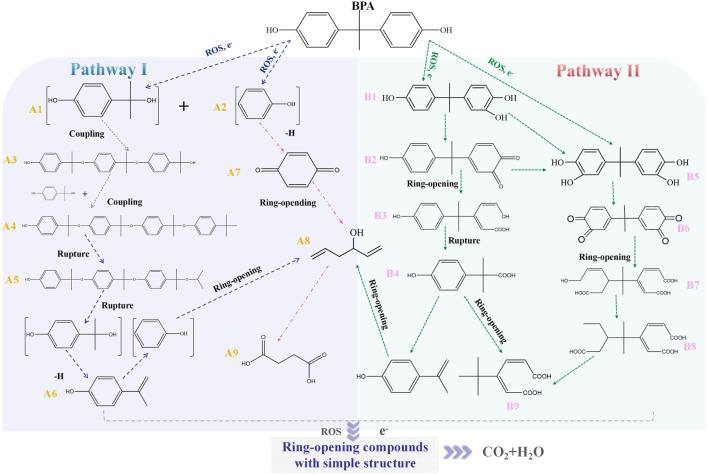
Proposed removal pathway of BPA in 3DER.

Accordingly, the removal pathway of BPA in 3DER is speculated in Figure 7. The C-C bond between two benzene rings was ruptured *via* ROS attack, resulting in part of BPA decomposing into A1 and A2. The A1 would couple with each other to form A3, A4, and possibly other coupling organics with relatively high molecular weight. Eventually, they transform into chain organics (A8 and A9) through C-C or C=C bone rupture and ring-opening reactions. Meanwhile, A2 could be transformed into 1,4-benzoquinone *via* dehydrogenated reaction before being oxidized to low molecular weight organics. On the other hand, the benzene ring(s) on the BPA molecule was attacked by water molecules, forming hydroxylated BPA (B1 and B5). Then the hydroxylated BPA was transformed into quinone (B2 and B6) and carboxylic (B3, B4, B7, B8, and B9) compounds through dehydrogenation and ring-opening reactions. Finally, the organics mentioned were oxidized into CO_2_ and H_2_O in 3DER. Many previous studies have supported a similar degradation process for aromatic compounds.

## 4 Conclusion

The major findings in this study are summarized as follows.

1) TMP was a micron-level material with good conductivity and catalytic ability, bearing Fe(II) and Fe(III). It could be used as PE to construct 3DER along with meshed stainless steel 2D electrodes.
2) The 3DER showed excellent performance for BPA removal under the optimal conditions: 200 ml of simulated wastewater, 9.00 initial pH, 300 mA current, and 15 mm electrode gap. Also, 1 ml of 0.5 M PMS and 1 g TMP were added to the system. Eventually, 98.1% of BPA was removed after 55 min of reaction under the optimal conditions.
3) The LC-MS results suggested two possible pathways for BPA degradation. BPA finally degraded to chain organics with simple molecular structure through C-C or C=C bone rupture and ring-opening reactions.

## Data Availability

The original contributions presented in the study are included in the article/Supplementary Material; further inquiries can be directed to the corresponding author.
